# Successful treatment with matched unrelated donor peripheral blood stem cell transplantation for very severe aplastic anemia in presence of active infections: A case report

**DOI:** 10.1097/MD.0000000000019807

**Published:** 2020-04-03

**Authors:** Yu-Rong Huang, Cai-Qin Xie, Jie-Feng Tong, Xiao-Hong Zhang, Yang Xu, Xiang-Gui Yuan

**Affiliations:** Department of Hematology, the Second Affiliated Hospital, Zhejiang University School of Medicine, Hangzhou, Zhejiang Province, China.

**Keywords:** active infection, matched unrelated donor, peripheral blood stem cell transplantation, severe aplastic anemia

## Abstract

**Rationale:**

Very severe aplastic anemia (vSAA) with active infections is always fatal. Adequate infection control before hematopoietic stem cell transplantation is recommended.

**Patient concerns:**

A 38-year-old woman with vSAA suffered from acute perforated appendicitis and invasive pulmonary fungal infection, and she failed to respond to intense antimicrobial therapies.

**Diagnosis:**

She was diagnosed with refractory vSAA with *stubborn* acute perforated appendicitis and invasive pulmonary fungal infection.

**Interventions:**

We successfully completed an emergent reduced intensity conditioning-matched unrelated donor (MUD)-peripheral blood stem cell transplantation (PBSCT) as a salvage therapy in the presence of active infections. The conditioning regimens consisted of reduced cyclophosphamide 30 mg/kg/day from day-5 to day-3, fludarabine 30 mg/m^2^/day from day-5 to day-3 and porcine-antilymphocyte immunoglobulin 15 mg/kg/day from day-4 to day-2 without total body irradiation. Cyclosporin A, mycophenolate mofetil and short-term methotrexate were administered as graft-versus-host disease (GVHD) prophylaxis. Neutrophils and platelets were engrafted on day+15 and day+21. Appendiceal abscess and severe pneumonia developed after neutrophil engraftment, which were successfully managed with intense antimicrobial therapy and surgical intervention.

**Outcomes:**

Only limited cutaneous chronic GVHD was observed 5 months after transplantation. The patient still lives in a good quality of life 2 years after transplantation.

**Lessons:**

Active infections may be no longer a contraindication to hematopoietic stem cell transplantation for some patients with vSAA.

## 1 Introduction

Severe aplastic anemia (SAA) is a disorder of stem cell failure, characterized by a failure of blood cell production with a marked hypocellular bone marrow, leading to refractory and prolonged pancytopenia. Matched sibling donor (MSD)-hematopoietic stem cell transplantation (HSCT) is the first-line approach for these patients younger than 35 years old.^[1]^ Matched unrelated donor (MUD)-HSCT may be considered when the patient is unavailable for a matched sibling donor and refractory to *immunosuppressive therapy*.^[1]^ Infections remain the major cause of death in SAA patients.^[2]^ HSCT for those patients with active infections generally has poor outcomes because of high infection-related and transplant-related mortality, and whats more, the presence of infection is an adverse factor for outcomes of HSCT.^[3]^ Adequate infection control before the transplantation is recommended.^[1]^ However, it may sometimes be necessary to carry on HSCT during active infections in the face of an otherwise inevitable death, because HSCT provides the best chance of early neutrophil recovery, which is of key importance for infection control.^[1]^ In this report, we successfully completed a MUD-peripheral blood stem cell transplantation (PBSCT) as a salvage therapy for a young patient with very SAA (vSAA) in the presence of active infections.

## 2 Methods

This study was approved by the Human Ethics Committee of the Second Affiliated Hospital, School of Medicine, Zhejiang University, China (the number of approvals: SAHZ-2018–036). Written informed consent was obtained from the family of patient for publication of this case report and any accompanying images.

## 3 Case report

**Figure 1:**
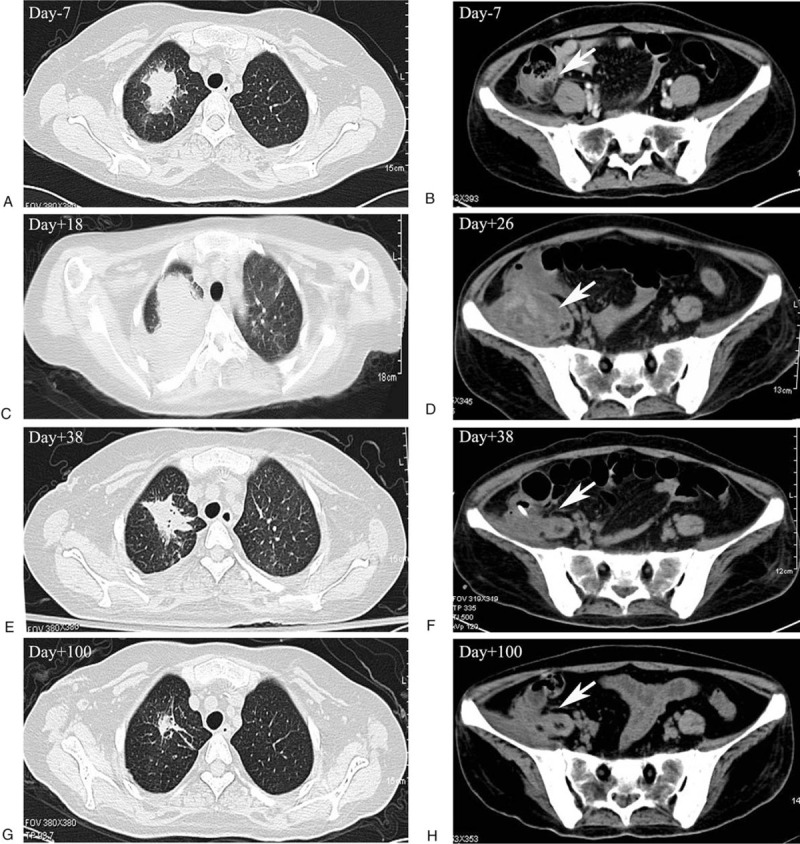
Dynamic images of acute perforated appendicitis and pneumonia before and after transplantation (A,C,E,G) Chest high resolution computed tomography images on day-7, day+18, day+38 and day+100 respectively, demonstrating the dynamic changes of the lesion in the right upper lung. (B,D,F,H) abdominal computed tomography images on day-7, day+26, day+38 and day+100, respectively, demonstrating the dynamic changes of acute appendicitis.

**Figure 2:**
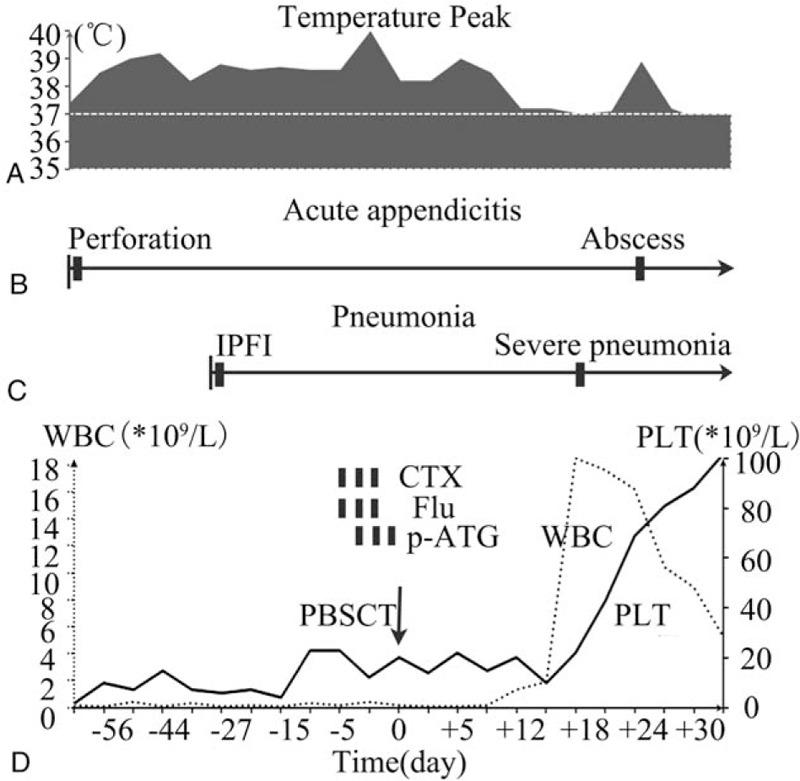
Clinical course associated with peripheral blood stem cell transplantation and active infections. (A) Temperature changes; (B) Clinical course of acute appendicitis; (C) Clinical course of pneumonia; (D) WBC and PLT changes; (Notes: WBC: white blood cell; PLT: platelet; CTX: cyclophosphamide; p-ATG: anti-human T lymphocyte porcine immunoglobulin; Flu: fludarabine).

A 38-year-old woman was diagnosed with vSAA in January 2017 according to the guidelines.^[1]^ She was unavailable for matched sibling donors (MSD) and refractory to the first course of *immunosuppressive therapy* (Cyclosporin A and Fresenius anti-human T lymphocyte rabbit immunoglobulin). To make matters worse, she suffered from persistent fever and abdominal pain 3 months after the diagnosis, and was later diagnosed with invasive pulmonary fungal infection and acute perforated appendicitis. She did not respond to the following 2 months of intense antimicrobial therapies (Fig. 1A, Fig. 1B). There were contraindications for appendectomy because of severe thrombocytopenia. At that moment, the general condition of the patient deteriorated with fatal neutropenia, transfusion dependence and persistent symptoms of fever and abdominal pain (Fig. 2). To achieve faster neutrophil recovery,^[4]^ we decided to perform an emergency peripheral blood stem cell transplantation (PBSCT) instead of bone marrow stem cell transplantation (BMSCT) 5 months after the first diagnosis of vSAA in the presence of *stubborn* active infections.

The day of stem cell re-infusion was termed as day0. The conditioning regimen consisted of cyclophosphamide 30 mg/kg/day from day-5 (5 days before stem cell re-infusion) to day-3, fludarabine 30 mg/m^2^/day from day-5 to day-3, and anti-human T lymphocyte porcine immunoglobulin (p-ATG, Wuhan Institute of Biological Products Company, China) 15 mg/kg/d from day-4 to day-2 without total body irradiation(TBI). Peripheral blood stem cells were transfused with 6.04 × 10^6^ CD34^+^ cells/kg(on day0). Cyclosporin A(CsA), mycophenolate mofetil (MMF) and methotrexate were administrated together as graft-versus-host disease (GVHD) prophylaxis. CsA(3 mg/kg/day) was gradually tapered 3 months later and discontinued 12 months after transplantation. MMF (20 mg/kg/day) was orally taken for 1 month. Methotrexate was injected at a dose of 15 mg/m^2^ on day+1(1 days after stem cell re-infusion), 10 mg/m^2^ on day+3, day+6, and day+11, respectively. Imipenem/cilastatin and voriconazole were employed for the anti-infection treatment during the transplant procedure.

Neutrophils and platelets were engrafted on day+15 and day+21, respectively. Fever subsided on day+15 after neutrophil engraftment. However, on day+18, chest tightness and shortness of breath with low oxygen saturation developed. The chest HRCT on day+18 revealed exacerbation of the pulmonary lesion (Fig. 1C). The antimicrobial agents were adjusted to caspofungin plus meropenem, which alleviated the symptoms soon and decreased the size of the pulmonary lesion (Fig. 1E). On day+26, fever and abdominal pain in the right lower quadrant were aggravated with a palpable large mass. The abdominal CT on day+26 indicated a periappendiceal abscess (Fig. 1D) in the right lower quadrant. Ultrasound-guided percutaneous drainage of the abscesses was performed and teicoplanin were added consequently to control the infection. Clinical symptoms were relieved after 2 weeks of treatment and the abscess volume decreased (Fig. 1F). Then, antimicrobial agents were adjusted to only oral voriconazole after discharge and both lesions resolved 3 months later (Fig. 1G, 1H). The clinical course associated with PBSCT and active infections was shown in Fig. 2.

The following course after transplantation was smooth and donor origin engraftment was confirmed by polymerase chain reaction analysis of DNA short tandem repeats. Only easy-control limited chronic skin GVHD was observed 5 months after transplantation. At the latest follow-up of 2 years after transplantation, the patient was still in complete remission and in a good quality of life.

## 4 Discussion

Matched sibling donor (MSD)-hematopoietic stem cell transplantation (HSCT) is the first-line approach for patients with SAA younger than 40 years old. For those patients without matched sibling donors, matched unrelated donors, mismatched donors including haploidentical donors or umbilical cord blood stem cells may be the alternatives.^[1]^ For HSCT in SAA, bone marrow stem cells (BMSC) are the preferred source in both matched sibling^[4]^ or unrelated transplantation^[5]^ due to less GVHD and better outcomes, compared with PBSC. However, similar to most transplant centers in China, we took PBSC other than BMSC because of faster engraftment and donor preference. Interestingly, Chen J reported that for patients with SAA in Asia Pacific area, PBSC was related to similar incidence of grade II-IV aGVHD (28·1% vs 17·4%), similar incidence of chronic GVHD (25·8% vs 29·3%) and similar overall survival (89·7% vs 82·4%), compared with BMSC.^[6]^ For our patient with active infections, PBSCT offered the faster neutrophil recovery, which is crucial for her infection control. Fortunately, with intensive GVHD prophylaxis regimens, our patient only experienced limited chronic GVHD, which was well controlled by the topical steroid therapy.

MUD-HSCT has higher rates of both acute GVHD and chronic GVHD than MSD-HSCT, although the outcome is currently similar.^[7]^ A key component of MUD-HSCT is the conditioning regimens. To date, the optimal conditioning regimens remain uncertain. EBMT(European Cooperative Group for Bone Marrow Transplantation) protocol comprises fludarabine (30 mg/m^2^ × 4), low dose cyclophosphamide (300 mg/m^2^ × 4) and ATG for 4 days, with a short course of both CsA and methotrexate as GVHD prophylaxis. The overall 2-year survival is 73% but graft failure is 18%.^[8]^ TBI was suggested to be integrated into the previous conditioning regimen with the 5-year overall survival rate of 79%, compared with 73% without TBI.^[9]^ No ATG in the conditioning is a strong negative predictor of survival.^[7]^ Alemtuzumab, another potent lymphocytotoxic immunosuppressant, has been trying to take the place of ATG in transplantation protocols.^[10]^ For our patient, in view of persistent active infections and poor general condition, we reduced the cyclophosphamide dose and shorted the duration of the conditioning to avoid severe toxicity, but we maintained a full dose of fludarabine and p-ATG (an alternative of Fresenius-rabbit-ATG in China) to avoid graft failure. We did not add TBI to the conditioning regimen, considering its significant early and late toxicities.

For vSAA with active infections, the outcome of HSCT is generally poor because of high infection-related mortality and transplant-related mortality. Aki^[11]^ reported 13 patients who had active invasive fungal infections with a median follow-up time of 306 days, only 4 patients (31%) survived the transplant procedure. Avivi^[12]^ reported 18 consecutive patients with a history of invasive fungal infections, only 1 patient of 5 (20%) with active infections survived the transplant procedure. For patients with active infections, broad-spectrum antibacterial, and/or antifungal agents are commonly suggested to completely control infections before HSCT. However, these attempts are usually of no avail. Anyway, it is sometimes necessary to proceed with HSCT regardless of active infections for the following reasons. Firstly, infection–related mortality markedly decreases due to earlier and improved diagnostic procedures as well as advances in infectious diseases supportive care.^[13]^ Secondly, with the recent advances in conditioning regimens, now we can minimize the degree of immunosuppression to reduce the opportunistic infections while ensure the engraftment. Finally, the transplantation offers the best chance of faster neutrophil recovery and infection control. HSCT has indeed saved a considerable proportion of recipients with active infections.^[14–17]^ Recently, a retrospective study stated that the outcomes of HSCT for SAA with pretransplant uncontrolled infections were not significantly different from that with controlled infection.^[18]^ A CIBMTR study demonstrated that although pretransplant invasive fungal infection is associated with slightly poorer outcomes after HSCT for hematological malignancies, pretransplant infection does not appear to be a contraindication to HSCT.^[3]^ For our patient, despite of broad-spectrum antibacterial and antifungal therapies, severe symptoms persisted and the poor general condition, HSCT was the best choice of treatment for faster neutrophil recovery and infection control.

In conclusions, we reported our experience on a young vSAA patient who was successfully treated with reduced intensity conditioning MUD-PBSCT as the salvage therapy during active infections. This suggests that MUD-PBSCT with a reduced-intensity conditioning regimen may be safe and effective for the treatment of vSAA with active infections. Further multicenter, large-scale and prospective studies are required to assess the safety and effectiveness of HSCT and its suitable conditioning regimen for the treatment of vSAA with active infections.

## Author contributions

**Conceptualization:** Yu-Rong Huang, Yang Xu.

**Data curation:** Yu-Rong Huang, Cai-Qin Xie.

**Funding acquisition:** Yang Xu.

**Investigation:** Yu-Rong Huang, Jie-Feng Tong, Xiao-Hong Zhang.

**Resources:** Cai-Qin Xie, Jie-Feng Tong, Xiao-Hong Zhang.

**Supervision:** Xiang-Gui Yuan.

**Writing – original draft:** Yu-Rong Huang, Xiang-Gui Yuan.

**Writing – review & editing:** Yang Xu, Xiang-Gui Yuan.

Xiang-Gui Yuan orcid: 0000-0003-0920-6171.
